# Adolescent pregnancy in Sao Tome and Principe: are there different obstetric and perinatal outcomes?

**DOI:** 10.1186/s12884-022-04779-9

**Published:** 2022-05-31

**Authors:** Alexandra Vasconcelos, Nelson Bandeira, Swasilanne Sousa, Maria Céu Machado, Filomena Pereira

**Affiliations:** 1 Unidade de Clínica Tropical ‑ Global Health and Tropical Medicine (GHTM), Instituto de Higiene e Medicina Tropical (IHMT), Universidade NOVA de Lisboa, Lisbon, Portugal; 2 Hospital Dr. Ayres de Menezes, República Democrática de São Tomé E Príncipe, Sao Tome, Sao Tome and Principe; 3 Faculdade de Medicina de Lisboa, Universidade de Lisboa, Lisbon, Portugal

**Keywords:** Antenatal care, Adverse pregnancy outcomes, Adolescent pregnancy, Sao Tome and Principe

## Abstract

**Background:**

Adolescent childbirth is a major public health problem in Sao Tome and Principe (STP). Adolescent pregnancy and childbirth can carry a risk of morbidity associated with the physiological and sociological characteristics of teenage girls. This study aims to identify the main adverse obstetric and perinatal outcomes for adolescent pregnancies in the Hospital Dr. Ayres de Menezes (HAM), the only hospital in STP.

**Methods:**

An institution-based cross-sectional study. Pregnant women ≤ 19 years of age (*n* = 104) were compared to non-adolescent women (*n* = 414). The obstetric and perinatal outcomes were compared between groups using the t test. Odds ratio (OR) were calculated through Cochran’s and Mantel–Haenszel statistics test for odds ratio equal to 1, 95% confidence intervals (CI) and p values (*p* < 0.05) were considered significant.

**Results:**

The adverse perinatal outcomes imputable to adolescent births were foetal distress with low first minute Apgar score < 7 (OR 1.94, 95% CI 1.18–3.18, *p* = 0.009) and performance of neonatal resuscitation manoeuvres (OR 2.4, 95% CI 1.07–5.38, *p* = 0.032). Compared to older mothers, teenage girls were likely to have a non-statistically significant threefold higher risk of having an obstructed labour (OR 3.40, 95% CI 0.89–12.94, *p* = 0.07). Other perinatal outcomes as neonatal asphyxia, risk for cerebral palsy, premature birth, early neonatal infection, and neonatal death were identical between groups as well as maternal anaemia, mode of delivery or other obstetrical outcomes.

**Conclusion:**

Adolescent pregnancies were associated with worse perinatal outcomes as foetal distress and higher need for neonatal resuscitation manoeuvres. This study may support STP health authorities in their efforts to make Sustainable Development Goals 3 (good health and wellbeing), 4 (quality education) and 5 (gender equality) a reality by 2030, since it identifies specific problems that need to be addressed to improve maternal adolescent health.

## Background

Adolescence is a stage of life that enables growth, development and behaviour optimization that sets a baseline for health in later life and for the next generation [1]. Adolescent girls (age ≤ 19 years at time of delivery) give birth to approximately 16 million babies each year, nearly 11% of births worldwide, being a global challenge linked with maternal and child morbidity and mortality that affects the socio-economic development of a country [2–5].

In Africa, adolescent pregnancies are a major public health issue as it is estimated that 33% of teenage girls give birth before the age of 18 and 3.5% even before the age of 15 years [2]. Low resource-constrained countries urge to reduce this public health imperative to reach the Sustainable Development Goals (SDG) by 2030 [6]. This is essential as adolescent pregnancies indirectly act as a drawback to the attainment of these goals [4] as they are at risk of socioeconomic hardship, poverty, out of school (Goal 1, 2 and 4) and to die from pregnancy-related complications (Goal 3) [4, 7–12].

Research suggests that adolescent girls compared to older women have higher risks of inadequate antenatal health care (ANC), cephalopelvic disproportion, obstructed labour, and death during pregnancy and childbirth [12–16]. Adolescent girl’s height and pelvic dimensions are only complete by two years after menarche, which can be linked to a higher risk for cephalopelvic disproportion and consequently to have an obstructed labour [14, 15]. To notice that obstructed labour is one of the most frequent and preventable causes of maternal and perinatal mortality and disability and a concern among teenage pregnancies [14].

Adolescent pregnancy can also be a risk to the baby [17–21]. There is substantial reason for arguing that adolescent´s newborns are more likely to be stillbirth, to die before they are one month old [21, 22], have low Apgar score [12, 21, 23], low birth weight (LBW) [21, 24] and to be admitted to an intensive care unit [21, 25].

Other adverse pregnancy outcomes are controversial in literature such as caesarean section, vaginal instrumental delivery, postpartum haemorrhage, and preeclampsia [26–30].

Some authors consider that adolescent child bearers are not a high-risk group if good ANC is provided [21, 31, 32] and Lawlor et al. [33] held that there is no biological reason to suggest that having a baby before the age of 20 is associated with higher risk for disability or mortality.

The evidence is controversial, as debate continues in whether the associations observed are related to the adolescent biological immaturity or were confounded by their lack of health care and frequently poor socioeconomic conditions [32–38]. Other factors have also been mentioned in the literature as explanations for this conflicting evidence, namely 1) heterogeneity of study settings (hospital-based vs population-based studies); 2) small sample sizes, mainly for adolescents < 15 years and 3) different conceptual approaches to adjust for potential confounders [32, 33].

Sao Tome and Principe (STP) is the second smallest sub-Saharan country but has one of the highest adolescent pregnancies prevalence in the region reaching around 27% [39]. There is, however, a knowledge gap concerning the obstetric and perinatal characteristics of this major public health problem in this country. The authors have also studied these pregnant adolescents´ characteristics and factors associated with early childbearing in STP, whose results were published in another article [40].

In the present study, we sought to identify the main adverse obstetric and perinatal outcomes for these adolescent pregnancies.

## Material and methods

### Study design

This is a cross-sectional analytical hospital-based study and data was collected among eligible study participants between July 2016 and November 2018.

### Study context

The study was conducted in Hospital Dr. Ayres de Menezes (HAM) in Sao Tome, the country´s capital, a growing city with 64% of the country population living in it [41, 42]. It is a high‐volume low‐resource hospital, with a maternity ward delivering around 82,4% of all the deliveries in the country with an average of 8 to 12 deliveries per day [39, 43].

The Democratic Republic of Sao Tome and Principe is a country with two islands in the Gulf of Guinea, in the western equatorial coast of Central Africa [39]. Women of childbearing potential represent about a quarter of the population and the number of expected pregnancies is around 5,000 per year [41–43]. The country´s maternal mortality ratio shows considerable progress from 158.3 maternal deaths per 100,000 live births to 74 per 100,000 live births in 2014, but the reduction was not enough to meet the Millennium Development Goal number five [41–43]. Antenatal health care is generally available in the country, with 98% of women being attended at least once by a skilled health provider during pregnancy [41, 42]. The coverage rate for at least four antenatal visits was 72% in 2009, rising to 84% in 2014 [41, 42]. STP level of poverty is high, with 66.2% of the population living on less than two euros per day, which is a major obstacle to achieve universal coverage [39, 41]. Antenatal consultations are free, but women pay around twenty euros for all mandatory routine tests. 

Comprehensive emergency obstetric care (caesarean sections and blood transfusions) is only available at the maternity ward of HAM, the only hospital in the country. High-risk patients are routinely referred to the HAM before, during labour, or after birth. At the maternity ward, the ratio of skilled midwives to deliveries is often one to three deliveries. In the daytime there are usually two obstetricians for performing the vaginal instrumental deliveries, caesarean sections, or other obstetric complications and on the night shift only one of them remains at HAM. Intrapartum foetal monitoring at HAM consists of a five-minute cardiotocography approximately every two hours and indication for restricted episiotomy during delivery depends on individual risk factors and obstetric conditions, as advocated by World Health Organization (WHO) [44]. Partograph and birth records are filled by the midwives. Neonatal resuscitation including suction, oxygen by bag and mask and cardiac massage follows the WHO standard procedures and is undertaken by the midwives. There is a Neonatal Care Unit (NCU) for term and premature newborns with six cots with no facilities for assisted ventilation.

### Study population and sampling

Participants were enrolled at HAM maternity ward after pregnant women admission for the baby´s delivery, there was a mean of 4540 deliveries during the study period.

Sample size was calculated using the WHO-steps approach [45] using a web based freely accessible sample size calculator, Raosoft® (http://www.raosoft.com/samplesize.html). Based on the formula for sample size and margin of error from the software, the minimum sample size was S = 355, which placed the right dimension between 355 (95%) and 579 (99%) confidence. For the original study, a total of 518 participants were enrolled for the study based on the following assumptions: two-sided 95% confidence level, power of 80% to detect an odds ratio of at least 2 for adverse birth outcomes. This sample size was also supported by PASS® software (https://www.ncss.com/software/pass/). Since, the sample size was not calculated for present outcomes, a power analysis was performed, varying from 63% to 87% for outcomes as neonatal resuscitation and foetal distress for this study.

The eligibility criteria for participants were: 1) prepartum women, 2) prepartum adolescents (under ≤ 19 years) who had obtained permission from their parent/s or legal guardian/s to participate in the study and 3) women and adolescents who gave birth outside the hospital but were admitted for postpartum care. The exclusion criteria included: 1) adolescents under 13 years of age; 2) adolescents who had not obtained permission from their parent/s or legal guardians to participate in the study; 3) adolescents with cognitive impairment; and 4) those admitted for abortion (gestational age < 28 weeks). None meet the exclusion criteria.

A random sampling was applied to recruit the study participants. Each morning, from the pile of mothers´ medical folders, we selected every second interval folder and then carry on asking for her consent for enrolment. The study was done at different months (two weeks every two other months) to avoid seasonal interference (rain season and malaria period), avoiding effects by means of confounding variables by guaranteeing a sample with few biases.

Five hundred and eighteen women aged 14–49 years consented to participate were included. The pregnant women were divided into two groups, ≤ 19 years old versus older > 19 years. Pregnancies that occur in a girl ≤ 19 years of age are referred to as an adolescent or teenage pregnancy. There were 104 pregnancies in women aged ≤ 19 years (adolescent group) and 414 in women more than 19 years (older women group). Participants (adolescents vs older counterparts) were also divided into primiparas (those having their first delivery) and multiparas (those having their second or above delivery) and the proportions of the complications were compared using Fisher’s exact test.

### Variables

In relation to the number of births they were classified as nullipara (para 0), primipara (para 1) or multipara (> para 1). ANC consisting of less than four consultations during pregnancy was considered as inadequate, between 4–7 as adequate and 8 or more visits as complete.

Gestational age was estimated from the date of onset of the last normal menstrual period, since the ultrasound dating of pregnancy is not usually done in this setting, although recommended during ANC consultations. Obstetric ultrasound was assessed in this study with the total number of scans per participant as well if performed before the 20^th^ week of pregnancy (early obstetric ultrasound).

Maternal outcomes investigated were: (i) anaemia during pregnancy (haemoglobin concentration < 11 g/dl), (ii) preeclampsia (hypertension ≥ 140/90 mmHg and proteinuria in dipsticks in women who were normotensive at ANC), (iii) perineal tear, (iv) episiotomy, (v) caesarean section, (vi) assisted  vaginal delivery (only performed by vacuum in HAM), and (vii) post-partum haemorrhage (> 500 mL bleeding). A diagnosis of pre-labour rupture of membranes (PROM) was made on clinical examination. Obstructed labour was operationally defined as the sum of all caesarean sections due to mechanical problems or foetal distress and all instrumental delivery.

Data about labour, delivery and its complications were collected from the partograph. Partographs tend to represent the monitoring of four parameters: 1) foetal heart monitoring, 2) uterine contractions, 3) blood pressure, and 4) pulse. A checklist was used to verify the correct use of partograph by the midwives (complete vs incomplete) in monitoring the four parameters mentioned above. We also review labour and delivery notes and supplemented with neonatal records.

The evaluated perinatal outcomes were: (i) prematurity, defined as a live infant delivered before 37 completed weeks of gestation from the date of onset of the last normal menstrual period, (ii) post-term labour, gestational period beyond 40 completed weeks as a prolonged pregnancy, (iii) low birth weight, newborn weight less than 2500 g at birth, (iv) large birth weight when ≥ 4000 g (macrossomia), (v) foetal death (dead infant after 28 weeks’ gestation) and (vi) admission at the Neonatal Care Unit.

Birth asphyxia was determined using the components of the APGAR score table [46], since techniques as umbilical arterial blood gas samples from a clamped section of the umbilical cord aren´t available in STP. The score comprised five components, which were appearance (colour), heart rate, grimaces (reflexes), activity (muscle tone), and respiration. A score of 0, 1 or 2 was given to each component. For this study the definition of (i) foetal distress was a low Apgar score < 7 at the first minute of life, (ii) clinical asphyxia was considered in infants with a 5-min Apgar score < 7 [47] and (iii) assessment of severity was defined as risk for cerebral palsy with an Apgar score of 5 or less at 5 min [46, 47]. This was similarly applied to both term and preterm infants.

### Data collection

Data on prepartum, intrapartum and postpartum characteristics of participants, were gathered and collected from 1) antenatal card, 2) obstetric maternal hospital and 3) newborn’s records. For prepartum data, relevant details of the perinatal history and antenatal period were collected systematically from the antenatal pregnancy cards. For intrapartum and postpartum data, mothers' and newborns’ medical hospital records were collected. Socio-demographic data were obtained through a face-to-face interview of the mothers, 12–24 h after delivery, using a structured questionnaire. The questionnaires were performed in Portuguese, the national language. The principal investigator (a paediatrician) executed and was responsible for all main activities as: 1) obtaining consent and enrolment of the participants, 2) data collection from antenatal cards plus maternal clinical and newborn’s records, 3) newborns` clinical exams (for diagnosis confirmation), 4) face-to-face interviews and 5) data collection entry in the database.

### Data management and statistical analysis

Data was secured in a confidential and private location. Participants were referred to by identification numbers and the informed consent forms were kept separate from the questionnaires. Both could only be linked by a coding sheet available only to the main investigator. Administered questionnaires were checked for completeness and consistency.

Data analysis in this study was carried out with the associations between maternal ages categorized as adolescent mothers (1) vs others (0). Each maternal and neonatal complication was assessed with Mantel = Haenszel Common Odds Ratio (OR) Estimate that was developed, resulting in the calculation of OR, 95% confidence intervals (CI) and p-values. For each outcome with a statistically significant difference at the first level, further analysis was developed using mothers´ age as a continuous variable with adjustment made simultaneously for the following factors, independently of their statistical significance: ultrasound and antenatal care. For statistical purposes, maternal age was kept as a continuous variable in the models. Mean age of the mothers was 26.59 years (sd 7.1) with a quasi-normal distribution, just underrepresented at the very young mother’s level. Two step logistic regression models were developed to assess the power of age as a predictor. The models were adjusted for marital status, education and antenatal care, the probabilities of event were recorded for each case and the plots obtained with the respective 95% CI for each age point.

Regarding confounding variables, multivariate logistic regressions were performed in two stages—first with only the variable under study (mother's age) and then including control variables to understand whether the identified effect occurred via other variables (education, presence of the partner and antenatal care).

Statistical significance was defined as p < 0.05. Data were analysed with SPSS software, version 26.0 (SPSS for Windows, Chicago, IL: SPSS Inc., 2020).

### Ethics approval and consent to participate

Approval to carry out this study was obtained from the Democratic Republic of Sao Tome and Principe Ministry of Health and Hospital Dr. Ayres de Menezes. Written informed consent was obtained from every participant (or their parent or legal guardian in the case of teenagers under 16) after the purpose of the research was explained orally by the investigator. Participation in the survey was voluntary, as participants could decline to participate at any time during the study. Anonymity and safety of participants were ensured. All methods were performed in accordance with the relevant guidelines and regulations in practice.

## Results

There were 104 pregnancies in adolescents (≤ 19 years) in 518 hospital deliveries and 414 in older women. The rate of adolescent pregnancy was 20.1% (CI – 16,3% to 23%), with a mean age of 17.42 years, median 17 with a minimum age 14 years old. Adolescent ages were: 1% (1) 14 years old, 7% (7) 15, 19% (20) 16, 24% (25) 17, 20% (21) 18 and 29% (30) 19 years old.

The older women group mean age was 28.9 years old, median 28 with a maximum age of 43. Regarding the adult group, the mother´s mean age at first pregnancy was 20.1 (± 3.76 SD) years (minimum 13, maximum 36).

### Intrapartum: antenatal characteristics and complications

Characteristics and complications in the antenatal period are described in Table 1. There was no maternal death in the study.

**Table 1:** Antenatal characteristics and complications

Characteristics	Adolescent pregnancies *n* = 104 (%)	Other pregnancies *n* = 414 (%)	OR	CI	*p* value
Primigravida*	73 (70.2%)	54 (13%)	15.7	9–26	≤ 0.001
Inadequate ANC (< 4 visits)	15 (14.4%)	55 (13.3%)	1.1	0.6—2	0.76
Preterm labour	10 (9.6%)	43 (10.4%)	0.98	0.4 – 1.9	0.82
PROM	9 (8.6%)	33 (7.9%)	1.1	0.5 – 2.4	0.83
Malpresentation	2 (2.2%)	6 (1.7%)	1.3	0.3—6.4	0.77
Past dates	13 (12.4%)	66 (15.3%)	0.77	0.4 – 1.5	0.42
Preeclampsia	10 (9.6%)	27 (6.5%)	1.55	0.71–3.26	0.27
Preeclampsia in nulliparous	7 (8.3%)	2 (2.9%)	3.0	0.60–14.94	0.18
Obstructed labour**	13 (12.5%)	42 (10%)	3.40	0.89–12.94	0.07
Anaemia	31 (42.5%)	135 (41.3%)	1.8	0.7—5	0.24
Rh incompatibility	3 (5.5%)	16 (5.7%)	1.05	0.3 – 3.7	0.94
IUGR	3 (2.9%)	17 (4.0%)	1.05	0.3—3.6	0.93

^*^ The proportion of primigravidas was higher in the adolescent group (OR 15.7, 95% CI 9–26, p ≤ 0.001) as compared to the older women as well as for nulliparous with 80.6% (83) in the first group and 16.3% (65) in the latter

^**^Compared to older mothers, adolescent mothers are likely to have a non-statistical significance of a threefold higher risk of having an obstructed labour

Abbreviations: *PROM *pre-labour rupture of membranes, *IUGR *Intrauterine growth restriction, *OR* Odds Ratio, *CI* Confidence interval

A first attendance at the antenatal care facility before the 12^th^ week of pregnancy was performed by 60.9% (63) of the adolescent group and by 52.8% (219) of the adult group.

The date of onset of the last menstrual period was not acknowledged by 8.7% (9) of the adolescents and by 12% (50) of the adult women group (OR 0.69, 95% CI 0.32–1.46, *p* = 0.335) which is considered not to be statistically significant.

There was no significant statistical difference between the two groups regarding the antenatal care: i) all adolescents (104) and (413) in the older women group were attended at least once by a skilled health provider during pregnancy; ii) inadequate ANC (0–3 visits) was observed in 14.4% (15) of the teenager group and 13.3% (55) in the older women (OR 1.1, 95% CI 0.6–2, *p* = 0.76); iii) adequate ANC (4–7 visits) was observed in 43.3% (45) of the adolescent group vs 47.3% (195); iv) complete ANC (> 8 visits) was verified in 42.3% (44) of adolescents and 39.4% (159) in older women; v) minimum of four visits in this pregnancy was observed in 85.4% (88) of the teenage group vs 86.4% (88).

One ultrasound scan was performed in 48.1% (49) of the adolescent pregnancies and 60.4% (248) in the control group (OR 0.61, 95% CI 0.4–0.9, *p* = 0.024). Two ultrasound scans were performed in 8.7% (9) of the adolescent pregnancy and 14.9% (59) in their older counterparts (OR 0.55, 95% CI 0.263–1.15, *p* = 0.376). Ultrasound scan before 20 weeks’ gestation (early ultrasound) was made in 41.7% (43) of the adolescents and 49% (98) in the older women group (OR 0.74, 95% CI 0.4–1.4, *p *= 0.30).

**Fig. 1:**
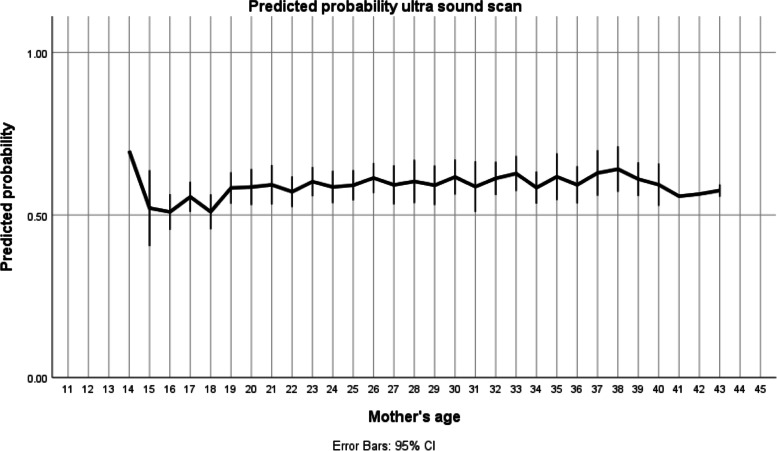
Probability of having an ultrasound during pregnancy according to mother´s age. * Analysis with age as a continuous variable. The model was adjusted for marital status, education, and antenatal care. The line at 0.5 represents equal probability of ultrasound

Although the analysis of adolescents versus older women seemed to point towards a difference in the probability of ultrasound during pregnancy that seems not to be the case when we extend the analysis of age as a continuous variable (Fig. 1). Most women have a probability slightly above 50%, while younger women seem to be just a little less likely to perform this exam. The variable age loses power to the control variable antenatal care. Women who are well followed during pregnancy have a higher probability of getting an ultrasound scan (OR 1.8, *p* = 0.004) independently of their age.

Comparison between primiparas and multiparas for both groups (adolescents vs older counterparts) revealed that the proportions of the complications were similar for both groups.

HIV and syphilis were not found in any adolescent compared to the adult group with 0.8% (4) and 1% (5) respectively. Malaria was diagnosed in 0.4% (2) of the teenage group vs 0.2% (1) in the older group. There is no statistically significant difference between groups.

### Intrapartum: mode of delivery and its complications

The mode of delivery and its complications are described in Table 2.

**Table 2:** Mode of delivery and its complications

Characteristics	Newborns from adolescents’ mothers *n* = 105 (%)	Newborns form older women *n* = 430 (%)	OR	CI	*p* value
Normal vaginal delivery	89 (84.8%)	361 (84%)	1.14	0.6–2.1	0.67
Caesarean section	12 (11.4%)	69 (16%)	0.675	0.351–1.3	0.24
Assisted vaginal delivery	4 (3.8%)	6 (1.4%)	2.8	0.8 – 10.1	0.11
Episiotomy*	17 (16.2%)	28 (6.5%)	2.8	1.5—5.4	0.002
Perineal tear	5 (5.5%)	9 (2.3%)	2.5	0.81 – 7.5	0.11
Postpartum haemorrhage	3 (2.9%)	8 (1.9%)	1.6	0.41 – 5.9	0.51

^*^When adjusted the predicted probability of episiotomy is reduced to near zero with age, with teenagers having a probability of about 0,2 (Fig. 2)

Abbreviations: *OR* Odds Ratio, *CI* Confidence interval

Home was the place of delivery for 4.1% (3) adolescents and for 2.5% (11) of the older pregnant (OR 1.663, 95% CI 0.5–5.4, *p* = 0.39).

Regarding the partograph register by midwives (during labour and delivery) both groups had an incomplete registration in 80% of the partographs. Midwives’ observations and monitoring were only recorded in a dismal 5% of cases, as per accepted frequency, as follows: foetal heart monitoring and uterine contractions 20%, blood pressure 14% and pulse 8%.

**Fig. 2:**
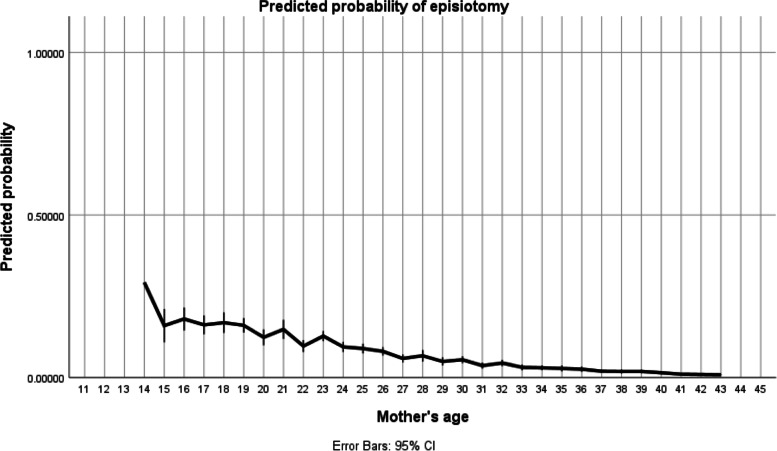
Predicted probability of episiotomy. *The model was adjusted for primiparas, marital status, education, and antenatal care. The line at 0.5 represents equal probability of episiotomy.

The practice of episiotomy was significantly higher in teenage pregnancies 16.2% (17) as compared to 6.5% (28) in the older women group (OR 2.8, 95% CI 1.5–5.4, *p* = 0.002) as observed in Table 2. Nevertheless, the probability is reduced to near zero with age, with young women having a probability of about 0,2 (Fig. 2) after adjusting for primiparas. The overall probability of episiotomy was generally low.

### Postpartum: perinatal outcomes

Perinatal outcomes are described in Table 3.

**Table 3:** Perinatal outcomes

Characteristics	Newborns from adolescents’ mothers *n* = 105 (%)	Newborns form older women *n* = 430 (%)	OR	CI	*p* value
Stillbirth	2 (1.9%)	14 (3.3%)	0.58	0.13 -2.6	0.47
Foetal distress*	30 (29.4%)	73 (17.7%)	1.94	1.18–3.18	0.009
Birth asphyxia	11 (10.9%)	31 (7.5%)	1.5	0.72–3.10	0.27
Risk for cerebral palsy	4 (3.9%)	9 (2.2%)	1.82	0.55–6.05	0.32
Neonatal resuscitation**	10 (9.5%)	18 (4.2%)	2.4	1.07–5.38	0.032
Infectious risk	19 (18,1%)	78 (18.1%)	0.99	0.6 – 1.7	0.99
Large gestational weight	1 (1%)	20 (4.7%)	-	-	-
Low birth weight	19 (18.3%)	81 (19.5%)	0.95	0.55–1.7	0.98
Congenital anomalies	3 (2.9%)	7 (1.6%)	1.8	0.5 – 6.9	0.41
Admission at NCU	15 (18.5%)	51 (13.9%)	1.4	0.74–2.64	0.29

^*^ There was a statistically significant difference between the two groups regarding foetal distress with low Apgar score (< 7) at the first minute of life, with 29.1% (30) in the adolescent group and 17.8% (74) in the older women group (OR 1.94, 95% CI 1.18–3.18, p = 0.009)

^**^ Neonatal resuscitation was performed more often in babies born from adolescent mothers 9.5% (10) compared to 4.2% (18) of the adult group (OR 2.4, 95% CI 1.07–5.38, *p* = 0.032)

Abbreviations: *NCU* Neonatal Care Unit, *OR* Odds Ratio, *CI* Confidence interval

Comparisons of pregnancy outcomes between very early (14–15 years), early (16–17 years) and late adolescence (18–19 years) found no significant differences. No difference was found in the comparison with women ≥ 35 years old. There was no significant difference between the two groups regarding other antenatal, delivery or newborn complications.

## Discussion

Pregnancy at an early age is regrettably common in many low-income countries and yet the debate about the degree of the associated maternal and perinatal risk is still open. Adolescent pregnancy is recognized as an important social and health concern in Sao Tome and Principe because it is often a trajectory in life steeped in social inequalities with adverse obstetric and perinatal outcomes [48, 49]. Through this study we aimed to identify if adolescent pregnant women had specific adverse outcomes when compared to older counterparts.

The adolescents age distribution findings revealed that those aged 14–15 years accounted for the least proportion (8%) of the study population, probably due to less developed secondary sexual characteristics in these younger teenagers. Still, this 8% rate of pregnancies in girls before the age of 15 years is two times higher to the 3.5% published for Africa [2]. On the other hand, almost 50% of the adolescent girls were aged 18 and 19 years old, which may be responsible for the few adverse outcomes identified in this study.

Most of the adolescents were primigravidas, what is expected, and which is also seen in similar studies [50]. Several studies have highlighted the importance of antenatal care in reducing the risks of adverse outcomes during pregnancy [21, 31–36]. The few adverse outcomes among adolescents reported in this study may be due to the high rates of ANC coverage in the country as all adolescents and older women were attended at least once by a skilled health provider during pregnancy. Comparing STP to other sub-Saharan African countries, the proportion of adolescent women who had accessed ANC at least once ranges from 29% in Ethiopia to 93% in South Africa [51].

A high rate of adolescents (85.4%) and older women (86.4%) had at least four antenatal visits and complete ANC were found in 42.3% of the adolescents, reinforcing a strong structure in ANC service available in the country. In other sub-Saharan countries this proportion drops to 30% in Ethiopia, 35% in Mali and in Guinea and 49% in South Africa [21].

Another positive aspect is that 60.9% of the adolescents had their first ANC attendance before the 12^th^ week of pregnancy contrasting to other studies [21, 50]. According to the WHO, in these countries, young pregnant women come late or not at all for antenatal care, mainly because of inadequate knowledge of its importance, poor family support and due to the fear of a reproving look of the caregiver on an adolescent who is pregnant [49–52].

In STP women who were well followed during pregnancy have a higher probability of getting an ultrasound scan independently of their age. However, it should be highlighted that almost half of the teenagers found the economic resource to do one ultrasound scan reinforcing the health education available.

The rate of obstetric complications in teenagers differs from study to study. In the present study, pregnancies in adolescents were not associated with a higher risk for preeclampsia but we found that young age and nulliparity had a threefold higher risk for preeclampsia, although with no-statistically significant difference (OR 3.0, 95% CI 0.60–14.94, p = 0.18), mainly due to the low number of patients enrolled.

It should be noticed that the delivery of the baby at home happened only in 4.1% of adolescents and in 2.5% of the older woman group. In STP despite limited specialized medical human resources of HAM, births attended by skilled health staff happened in 93% of cases, considerably higher than in other Portuguese-speaking countries, namely 91% in Cape Verde, 68% in Equatorial Guinea, 50% in Angola, 54% in Mozambique and 45% in Guinea-Bissau [53].

Regarding childbirth, some authors suggest that the physical immaturity of the adolescent girl, especially her pelvis, is a factor of dystocia [14, 15]. Similar to our findings, other studies did not find any increase in the caesarean section rates among adolescents [50]. Episiotomy appeared more likely to be done among the adolescent group, possibly due to their developing and rigid perineum but when the model was adjusted, this rate was reduced to near zero. There were also low rates of perineal tear at vaginal delivery in both groups, with a tendency of a twofold higher risk for teenagers (OR 2.5, 95% CI 0.81–7.5, *p* = 0.11) but also with no-statistically significant difference. These data suggest that a better perineal guard in labour at HAM should be thought to minimize perineal tear.

There were no maternal deaths in this study. In STP, there were three cases of maternal deaths reported during the study period, from which two were in the 15–19 age group (adolescent group) and one in the group of 25—29 years [42]. Regarding to 2017 data on maternal mortality rate (MMR), Cape Verde estimates only 43 maternal deaths per 100,000 live births, while STP maintains 73 MMR. Nonetheless, STP´s MMR is lower when compared to the other countries such as Guinea-Bissau with 457, Mozambique 206, Equatorial Guinea 181, and Angola 167 MMR [53].

There was no statistically significant difference in stillbirths born to adolescents or older mothers. This was consistent with findings from other studies where adolescents were just as likely to have stillbirths as older women [2]. In the present study, neonates from adolescent mothers were similarly born at term as those from their adult counterparts. This finding is contrary to that observed by some authors [8, 12, 54, 55].

We have not found any statistically significant difference concerning congenital malformations in newborns of adolescent girls contrarily to Vinatier et al. [56] that reported more malformations in newborns of mothers under age 15 (21%), than in those from older women.

Our results are opposite to most literature, which show that adolescent pregnancies have poorer neonatal birth outcomes, as prematurity, small for gestational age and stillbirth and neonatal deaths [26–30]. We did not find an association between young maternal age and neonatal morbidity or mortality except for foetal distress and neonatal resuscitation.

Foetal distress and higher need for neonatal resuscitation manoeuvres found in the adolescent group reflect a difficult transition between in-utero to life. This can be probably due to the biological concern that younger women are more likely to have an immature pelvis, as it continues to grow throughout adolescence. This can lead to cephalopelvic disproportion, obstructed labour, or other obstetric complications [15].

We found a weak statistically significant difference (*p* = 0.07) in the adolescent group for a threefold higher risk of having an obstructed labour. This was consistent with results from other studies carried out in Cameron and in other sub-Saharan African countries [8].

Regarding assisted vaginal delivery, we found a twofold higher risk of it being performed in the adolescent group, but also with a no-statistically significant difference (*p* = 0.11), mainly due to the low number of participants enrolled.

This highlights the need for specialist attention for adolescent mothers’ labours and a prompt referral to a caesarean section when needed. These obstetric complications can also be reduced by using the partograph to assess the labour progress. Foetal monitoring may provide crucial information on the adequacy of foetal oxygenation during labour for timely and appropriate management of complications. Even though we have found that the partograph sheet was available in all obstetrical records, accurate recording of parameters—to monitor the foetus, the mother, and the progress of labour—was rarely done by the midwives. From our field experience, we can speculate or predict that interventions, such as advocacy for the use of the partograph should be encouraged at the maternity ward of HAM and probably in many other sub–Saharan African hospitals for prevention of foetal distress.

Furthermore, the emotional and psychological needs of pregnant adolescent girls can be greater than those of older women [52]. At HAM there is a complete isolation of the labouring woman from family members, which is unusual in most contemporary labour settings [44, 49]. This factor can worsen the adolescent preparedness to cooperate during the baby’s birth. In our opinion, service planners need to offer every pregnant woman privacy, allowing her to be accompanied by a supportive person throughout labour and delivery. An attractive and humanistic setting should be provided since this approach reduces the need for medical interventions and has the potential to encourage greater use of health facilities [22, 44, 52].

Nonetheless, newborns from adolescent mothers did not experience more risks for birth asphyxia nor for cerebral palsy.

### Strength and limitation of the study

This study has several limitations. Firstly, the estimation of adverse risks could be biased, as the study was implemented in the country´s only health facility with the capacity to perform caesarean sections. Secondly, the study was carried out in one health facility, in the country’s capital, and cannot be used for generalisations for the rest of the country. The results obtained from adolescent deliveries underestimates the burden of adolescent pregnancies in Sao Tome and Principe, as several girls may have had abortions or may have given birth at home and could therefore not be recruited into the study.

Nevertheless, this study allowed us to build the first map-making of the adolescent deliveries situation in Sao Tome and Principe which could help in policy-making and clinical decisions.

This study included a total number of pregnant women similar to the one published by UNICEF in the Multiple Indicator Cluster Survey (MICS) for STP [39]. In contrast to MICS, our study has the advantage of not being vulnerable to memory or recall bias because the data were gathered for the current pregnancy and delivery with data abstracted directly from clinical records and antenatal cards.

### Future studies

A future study with a superior number of participants could clarify if the higher tendencies identified in adolescents regarding obstructed labour, assisted vaginal delivery, and preeclampsia in nulliparous are statistically significant, in order to plan a successful intervention.

### Contribution of our study to knowledge

No study on this subject has previously been published on the risk factors and the maternal and perinatal prognosis of childbirth in adolescents in Sao Tome and Príncipe. It is the first comprehensive study of this problem in the country, integrating a multivariate analysis to assess maternal and perinatal outcomes.

## Conclusions

Adolescents had adverse perinatal outcomes as foetal distress and a higher need for resuscitation manoeuvres of their newborns. Compared to older mothers, adolescent mothers are likely to have a non-statistical significance of a threefold higher risk of having an obstructed labour.

We can also guess the existence of other than age-related factors for foetal distress in adolescent mothers, namely emotional and psychological issues, and difficulties of an unprepared girl to face the labour stages and cooperation during the baby’s delivery. Therefore, pregnancies and deliveries among adolescents should be carefully monitored.

The good antenatal care coverage rates shown can be accountable for the few adverse outcomes found for adolescent pregnancies in this study.

To sum up, this study provides a baseline data on the complications among pregnant adolescents in this low resource country and may contribute to the local efforts to make SDG for maternal health a reality by 2030.

## Data Availability

The datasets used and/or analyzed during the current study are available from the corresponding author on reasonable request.

## Abbreviations

STP: Sao Tome and Principe
HAM: Hospital Dr. Ayres de Menezes
OR: Odds Ratio
CI: Confidence interval
SDG: Sustainable Development Goal
ANC: Antenatal care
MICS: Multiple Indicator Cluster Survey
WHO: World Health Organization
PROM: Pre-labour rupture of membranes
IUGR: Intrauterine growth restriction
NCU: Neonatal Care Unit
